# CORRELATION BETWEEN PRE AND POSTOPERATIVE UPPER DIGESTIVE ENDOSCOPY IN PATIENTS WHO UNDERWENT ROUX-EN-Y GASTROJEJUNAL BYPASS

**DOI:** 10.1590/0102-6720201600010009

**Published:** 2016

**Authors:** Leticia Elizabeth Augustin CZECZKO, Manoela Aguiar CRUZ, Flávia Caroline KLOSTERMANN, Nicolau Gregori CZECZKO, Paulo Afonso Nunes NASSIF, Alexandre Eduardo Augusto CZECZKO

**Affiliations:** Postgraduate Program in Principles of Surgery, Evangelic Faculty of Paraná/University Evangelic Hospital of Curitiba/Medical Research Institute, Curitiba, PR, Brazil

**Keywords:** Bariatric surgery, Roux-en-Y gastrojejunal bypass, Endoscopy, Obesity

## Abstract

**Background:**

Bariatric operations have variable range of complications and postoperative benefits. Gastroesophageal reflux is considered potential factor that may result in damage to the esophageal mucosa and this subject is quite controversial in the literature.

***Aim*:**

To evaluate patients who underwent to Roux-en-Y gastrojejunal bypass correlating epidemiologic and endoscopic findings in pre and postoperative periods.

***Method*:**

A retrospective, paired study which evaluated 110 patients. Inclusion criteria were formal indication for bariatric surgery and patients with pre and postoperative endoscopy. Exclusion criteria were previous bariatric surgery, patients subjected to other types of bariatric surgery and those who had no pre or postoperative upper digestive endoscopy. The epidemiological variables were: sex, age, body mass index, type 2 diabetes mellitus or impaired glucose tolerance, and preoperative dyslipidemia.

***Results*:**

The preoperative upper endoscopy was normal in 26.4% of the patients. Among endoscopic alterations, the hiatus hernia was the most prevalent followed by non-erosive gastritis. The postoperative upper endoscopy was normal in 40.9% and stenosis was the most prevalent followed by marginal ulcer. Correlation on pre and postoperative endoscopies, was found 100% reduction of hiatal hernias and 88% of esophagitis. There was no statistical significance in relationship to anastomotic stenosis with preoperative other variables.

**Conclusions:**

There was significant decrease in postoperative hiatus hernia, erosive esophagitis, non-erosive esophagitis, erosive gastritis and non-erosive gastritis with the operation. Stenosis of the gastrojejunostomy anastomosis was the most prevalent postoperative complication with no correlation with preoperative variables.

## Abstract (pt)

**Racional:**

As operações bariátricas apresentam gama variável de complicações e benefícios pós-operatórios. O refluxo gastroesofágico é considerado potencial fator que pode produzir lesões na mucosa esofágica e este tema é bastante controverso na literatura.

***Objetivos*:**

Correlacionar dados epidemiológicos e sua relação com achados endoscópicos no pré e pós-operatórios de pacientes submetidos ao bypass gastrojejunal em Y-de-Roux.

***Métodos*:**

Estudo retrospectivo, pareado no qual foram avaliados 110 pacientes. Os critérios de inclusão foram indicação formal à cirurgia bariátrica e possuir endoscopias digestivas altas do pré e pós-operatório. Foram excluídos pacientes submetidos a operações bariátricas prévias; a outros métodos cirúrgicos bariátricos; que não possuíssem endoscopia digestiva alta pré e pós-operatórias. As variáveis epidemiológicas estudadas foram: sexo, idade, IMC, diabete melito tipo 2 ou tolerância à glicose diminuída, e dislipidemia no pré-operatório.

***Resultados*:**

O sexo feminino foi prevalente em 73,6% da amostra, a idade média de 37,3 anos; o IMC foi de 40,3 kg/m^2^. O diabete melito tipo 2 ou tolerância à glicose diminuída foi diagnosticada em 38,2% dos pacientes e a dislipidemia em 53,6%. A endoscopia digestiva alta pré-operatória foi normal em 26,4%. A hérnia de hiato foi alteração mais prevalente, seguida pela gastrite não erosiva. No pós-operatório a endoscopia foi normal em 40,9%, a estenose da anastomose gastrojejunal foi a alteração mais prevalente, seguida da úlcera marginal. Correlacionando-se as endoscopias pré e pós-operatórias encontrou-se redução de 100% das hérnias de hiato e 88% das esofagites. As demais variáveis não apresentaram significância estatística.

***Conclusões*:**

A correlação entre os achados pré e pós-operatórios das endoscopias digestivas altas indicou diminuição no pós-operatório de hérnia hiatal, esofagite erosiva, esofagite não erosiva, gastrite erosiva e gastrite não erosiva. A estenose da anastomose gastrojejunal foi a complicação pós-operatória mais prevalente sem correlação com as variáveis pré-operatórias estudadas.

## INTRODUCTION

Obesity is considered a public health issue in modern society, and its prevalence has increased both in developed and developing countries. With the higher number of obese individuals, it is possible to observe a greater incidence of metabolic syndrome, cardiovascular diseases, gastroesophageal reflux disease and obstructive sleep apnea, all of those concomitant to the anatomic alterations associated with weight excess^1^. These comorbidities per se, along with atherosclerotic disease, produce a reduction of seven years (female) and nine years (male) in the life span of obese individuals, particularly the ones in the third stage^2.^

Roux-en-Y gastrojejunal bypass is the most frequently performed bariatric surgery in Brazil and worldwide. It presents, in general, more effective weight loss than the adjustable gastric banding of Scopinaro procedure^3^.

It is recommended that all bariatric procedure should be done by a multidisciplinary team^4^. In this group, should be include the endoscopist who work in the preoperative and postoperative periods, helping both to diagnose and to define therapy for possible complications. There is no unanimity in the literature according to when and who should undergo upper digestive endoscopy on the preoperative and postoperative periods. Some authors defend that they should be performed in selected symptomatic patients, while others believe that it should be done in all patients. 

Thus, considering the doubts which still exist about the real necessity to perform upper digestive endoscopy on the preoperative and postoperative periods of bariatric surgery and clarify the positive and negative relations among the endoscopic findings and the surgical procedures, it is important to perform studies in order to elucidate these questions and reduce morbidity in bariatric surgery.

Thereby, this study aims to assess the clinic/endoscopic correlation of epidemiologic and endoscopic data collected from the surgical procedure in a sample of obese patients who underwent Roux-en-Y gastrojejunal bypass. 

## METHOD

Was performed a retrospective paired follow-up study, with a sample of 110 individuals who underwent Roux-en-Y gastrojejunal bypass between January 2011 and July 2012.

The criteria for inclusion were: 1) patients with obesity who had indication of surgery determined by the parameters stablished by the Brazilian Society of Bariatric and Metabolic Surgery and clinical intractability of obesity determined by endocrinologist; and 2) patients who underwent Roux-en-Y gastrojejunal bypass either through laparotomic or videolaparoscopic technique by the same surgeon.

The criteria for exclusion were: 1) patients who had undergone previous bariatric surgery; b) individuals who underwent other types of bariatric procedure; c) patients who did not have preoperative and postoperative upper endoscopy. 

Were collected name, gender, age, date of surgery, body mass index, presence of diabetes mellitus type 2 or reduced tolerance to glucose, dyslipidemia and the results of the preoperative and postoperative upper endoscopy. 

Upper gastrointestinal endoscopy was divided into normal and altered. Among pathological findings, we considered for preoperative: hiatal hernia, erosive esophagitis, non-erosive esophagitis, Barrett's esophagus, esophageal gastric ectopy, esophageal papilloma, eosinophilic esophagitis, single or multiple polyps, erosive gastritis, non-erosive gastritis, nodular gastritis, duodenitis, duodenal ulcer and cancer. For postoperative were considered: hiatal hernia, erosive esophagitis, non-erosive esophagitis, erosive gastritis, non-erosive gastritis, marginal ulcer, non-marginal ulcer, stenosis of the gastrojejunal anastomosis, food impaction, residual gastric fundus and gastrogastric fistula.

The results of quantitative variables were described through means, medians, minimum values, maximum values and standard deviations. Qualitative variables were described through frequencies and percentage. In order to compare the assessment moments to the dicotomical variations, was considered the binomial test. The assessment of the association between two qualitative variables was performed with the Fisher's exact test. To compare two groups defined through qualitative variables in relation to the quantitative ones, was considered the Student's t-test for independent samples. The conditions for normality of the variables was assessed through the Kolmogorov-Smirnov test. Values of p<0.05 indicate statistical significance. Data were analyzed with the software IBM SPSS Statistics v.20.

## RESULTS

Among the 110 patients, 29 (26.4%) were male and 81 (73.6%) female. The average age was 37.3 years old with an average 37.9 years old for female and 35.4 for men, with no relevant difference (p=0.272). 

The distribution according to the stages of BMI in the preoperative period was: stage 1 for six; stage 2 for 47 and stage 3 for 57 patients. Average BMI for men was 41.3 for male and 39.9 for women with no relevant difference (p=0.197).

In the sample, 42 patients (38.2%) had diabetes or reduced tolerance to glucose in the preoperative period and 68 presented no hyperglycemic disorder. There was no relevant statistic difference in the comparison between male and female patients (p=0.504). 

Among the 110 patients, 59 presented criteria for dyslipidemia in the preoperative period (53.7%) while 51 did not. Comparing male to female, there was no relevant difference (p=0.831). 

Observing the preoperative upper endoscopy evaluation, 29 patients had normal exams. In 81 patients, pathologic findings were detected, and 50 patients had more than one diagnosis. Table 1 shows these results.

**TABLE 1:** - Preoperative endoscopic findings

**Preoperative endoscopic diagnosis**	**n (%)**
Normal	29 (26.4%)
Altered	81 (73.6%)
Hiatal hernia	36 (32.7%)
Erosive esophagitis	25 (22.7%)
Non-erosive esophagitis	29 (26.4%)
Barrett's esophagus	3 (2.7%)
Esophageal gastric ectopy	2 (1.8%)
Esophageal papilloma	1 (0.9%)
Eosinophilic esophagitis	1 (0.9%)
Single or multiple polyps	5 (4.5%)
Erosive gastritis	17 (15.5%)
Non-erosive gastritis	30 (27.3%)
Nodular gastritis	3 (2.7%)
Duodenitis	5 (4.5%)
Duodenal ulcer	1 (0.9%)
Cancer	0 (0.0%)

Note: Altered findings add more than 81 because there were patients with more than one endoscopic alteration

In the postoperative period 45 patients had normal exams. In 65 the endoscopy presented some pathologic finding (Table 2)

**TABLE 2:** - Postoperative endoscopic findings

**Postoperative endoscopic diagnosis**	**n (%)**
Normal	45 (40.9%)
Altered	65 (59.1%)
Hiatal hernia	0 (0.0%)
Erosive esophagitis	0 (0.0%)
Non-erosive esophagitis	5 (4.5%)
Erosive gastritis	0 (0.0%)
Non-erosive gastritis	6 (5.5%)
Non-marginal ulcer	4 (3.6%)
Surgical Complications	
Marginal ulcer	9 (8.2%)
Stenosis of gastrojejunal anastomosis	39 (35.5%)
Food impaction	2 (1.8%)
Residual gastric fundus	2 (1.8%)
Gastrogastric fistula	3 (2.7%)

Note: Altered findings add more than 65 because there were patients with more than one endoscopic alteration

Comparing normal and altered preoperative and postoperative endoscopies, there was a significant statistic difference (p=0.019) with a change from altered in the preoperative to normal in the postoperative. 

Out of 110 patients, 36 (32.7%) had hiatal hernia in the preoperative and did not have it anymore in the postoperative. The correlation between these data is statistically significant (p<0.001).

In the sample, 25 patients had erosive esophagitis and 29 had 29 non-erosive esophagitis in the preoperative period. In the postoperative period, there were no patients with erosive esophagitis and five were diagnosed with non-erosive esophagitis. The comparison of these data was statistically significant (p<0.001). 

There were 17 patients with erosive gastritis and 30 with non-erosive gastritis in the preoperative period. In the postoperative period there where no patients with erosive gastritis and six were diagnosed with non-erosive gastritis. The comparison of these data was statistically significant (p<0.001).

**FIGURE 1:**
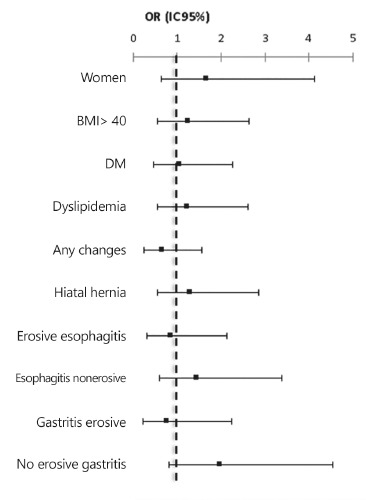
- Odds Ratio for postoperative stenosis, considering demographic variables and endoscopic alterations evaluated in the preoperative period

The main surgical complication was the stenosis of the gastroenteric anastomosis. Was compared preoperative individual and endoscopic factors with the findings of postoperative stenosis. Was not found any statistically significant relation with the analyzed variables (Figure 1).

## DISCUSSION

### Preoperative upper endoscopy

Many authors defend preoperative upper endoscopy for all candidates to surgery^5^, while others state that it should be performed only in patients with some gastrointestinal complaint^6^. The main reason for whom defending that it should not be a routine exam is that it only changes the surgery, the technique or the type in the minority of the patients^7^. 

To clarify the importance of the endoscopic results, Sharaf et al., stablished a division of the preoperative endoscopic findings into four groups: group 0=normal; group 1=altered exams which do not change/postpone the surgery (as esophagitis, gastritis and/or mild duodenitis, esophageal membranes); group 2=results that change or postpone the surgery (mass lesions-mucosa/submucosa, ulcers in any location, esofagitis, gastritis and/or severe erosive duodenitis, Barrett's esophagus, bezoar, hiatal hernia,peptic stenosis, esophageal diverticula, arteriovenous malformation, Zenker's diverticula); and group 3=absolute contraindications to surgery (upper gastrointestinal cancer and varices)^8^. Out of 195 upper endoscopies performed 10.3% was in group 0; 28.2% in group 1; and 61.5% in group 2. Having found high rates of clinically important lesions, it can be stated that routine upper endoscopy has a good cost-benefit before the bariatric procedure. 

On the other hand, the proposal made by Loewen et al. modifies the classification created by Sharaf et al. and includes hiatal hernia in group 1, obtaining only 18% of patients with a change over the timing, technique or contraindication to surgery^8^^,^ 9. They judge that the usage of preoperative upper endoscopy is questionable. According to the author's opinion, the presence of hiatal hernia in the preoperative period modifies the surgery technique that has been planned. It already occurs in the service of bariatric surgery at the initial indication for vertical gastrectomy and gastric banding. Tariq e Chand proposed a new classification that describes in a more detailed way the diagnoses of the four groups^4^.

In the preoperative period of this study, only 29 patients (26.4%) presented normal endoscopy, while in the postoperative this number increased a little, but still representing the minority of patients, 45 cases (40.9%). As most of them had pathological endoscopic findings and needed treatment, it is evident the importance of the routine preoperative endoscopic exam when there is an indication to bypass. 

Preoperative endoscopic alterations were found in 81 patients (73.63%). Almeida et al. and KÜPER et al. (2010) presented similar results with 77.2% and 79.7%, respectively^10^^,^ 11. There are studies with higher rates, as the one by Madan et al., that reported gastrointestinal abnormalities in 91% of the sample; and lower rates, as in the study by Csendes et al. with 10%, Korenkov et al. with 33.9% and Dietz et al. with 57.9%. It demonstrates the influence of certain epidemiologic aspects, individual habits and food habits in different regions, populations and continents^12^^,^ 13 ^,^ 14 ^,^ 15.

Among the preoperative endoscopic diagnoses, hiatal hernia was preponderant, prevailing in 31.1% of the patients. A similar result was found by Küper et al.(2010) with 27.5% and Dietz et al. with 24.6% but with hiatal hernia in second place, with erosive gastritis as the first (30.2%)^15^. Loewen et al. found the presence of hiatal hernia in 9% of the patients and non-erosive gastritis as the main finding, in 13.6%^9^. Almeida et al. also found non-erosive gastritis as the main finding in 50.7% of the patients and 8.6% presented hiatal hernia^10^. Muñoz et al. had gastritis as the most relevant preoperative finding, in 21% of the sample and hiatal hernia present in 10.7% of the sample^16^. Bahra et al. found as the main alterations gastritis and hiatal hernia in 13.6% each^6^.

If is added up the diagnoses of erosive (n=17) and non-erosive (n=30) gastritis this study presents a rate of 42.8%, similar to the others authors^9^^,^ 15 ^,^ 16. 

### Proposal for endoscopic classification pre Roux-en-Y gastrojejunal bypass 

The classification of endoscopic findings set during the preoperative period of bariatric surgery shows the importance of preoperative screening. As the previous classifications refer to any type of bariatric surgery, the authors of this study believe they do not match this research assessing only patients who underwent Roux-en-Y gastrojejunal bypass. The authors of this paper propose a new specific classification for preoperative endoscopic findings before Roux-en-Y gastrojejunal bypass, as it follows: 

Group 1: Normal endoscopic findings; 

Group 2: Findings that do not postpone the surgery, but demand specific care (hiatal hernia with reflux symptoms, mild esophagitis, mild gastritis, mild duodenitis (clinical treatment) and non-dysplastic polyps (polypectomy); 

Group 3: Finding that postpone the surgery with the necessity of endoscopic revision (peptic ulcer, erosive esophagitis, erosive gastritis, Barrett's esophagus with confirmed no dysplasia, multiple polyps, dysplastic polyp - excluded stomach must be resected or vertical gastrectomy must be performed); 

Group 4: Contraindication for Roux-en-Y gastrojejunal bypass: esophagogastroduodenal malignancy, Barrett's esophagus with upper stages of dysplasia, esophageal varices, severe gastric polyposis.

### Postoperative upper endoscopy

During the postoperative period, endoscopy is usually required in face of symptoms; because of that, most studies are performed on symptomatic patients and these studies aim to relate the clinical conditions to the endoscopic findings^17^^,^ 18 ^,^ 19 ^,^ 20. At the unit where this research was developed, the requirement for endoscopy is when there is upper gastrointestinal symptoms or after a year of surgery for patients with no symptoms.

Was found 45 patients with normal endoscopy and 65 (59.1%) with alterations. Rocha put together symptomatic (85.3%) and asymptomatic (14.7%) patients and presented alteration in 35.8% of his endoscopies^21^. Yang et al. used symptomatic sample and found alteration in 77.6% of the endoscopies^18^. Wilson et al. and Lee et al. also required endoscopy only for symptomatic patients and found results similar to this study, with 56% and 68.4% of alterations in endoscopies after Roux-en-Y gastrojejunal bypass^19^^,^ 20.Contrary to other studies, Spinosa and Valezi evaluated only asymptomatic patients after one year of surgery and found endoscopic alterations in only 26.5% of the sample in the postoperative period. They concluded that upper endoscopy is important even for asymptomatic patients^22^.

The greatest disagreement among the findings of postoperative upper endoscopy occurs precisely because symptomatic patients have a far greater predictive factor for alterations, compared to asymptomatic^23^. In this study, were selected patients from continuous sample, regardless of the postoperative outcomes, including symptomatic and asymptomatic. Thus, was found a series which is less biased in relation to the postoperative endoscopic findings.

### Pre and postoperative endoscopic correlation

In the correlation between pre and postoperative endoscopies, hiatal hernia presented a significant result (p<0.001) according to its disappearance after the surgery. Was observed that the conclusive reason for this fact is the surgical technique, associated to the weight loss caused by the procedure. Such loss leads to reduction of intra abdominal pressure and consequent reduction of the herniation of the stomach portion to the posterior mediastinum. A similar result was found by Cardoso, who obtained a reduction of 100% of the hiatal hernias^24^.

The correlation between the pre and postoperative findings for gastroesophageal reflux disorder was statistically significant (p<0.001). The analysis of the endoscopic findings for erosive and non-erosive esophagitis indicates a reduction of 100% for the first and 88% for the second. Similar findings were observed in a study that also paired pre and postoperative endoscopy by Cardoso who obtained 75% reduction of reflux esophagitis^24^.In the correlation between pre and postoperative endoscopic findings, the reduction of 100% erosive gastritis and 89% non-erosive gastritis in the postoperative period was statistically significant (p<0.001). The surgical protocol of authors team prescribes the routine usage of proton-pump inhibitor (omeprazol 20 mg) in the preoperative period and if there are peptic endoscopic alterations, for two months in the postoperative period. 

Among the alterations after Roux-en-Y gastrojejunal bypass, the gastrojejunal anastomosis was the most prevailing postoperative complication. It occurred to 39 patients (35.45%), as well as in the study by Lee et al. in 52.6%^20^. Ramos et al., who assessed 12,000 patients, found 303 cases of stenosis (2.5%)^25^. These authors comment that the rates of stenosis decreased to 0.8% after the introduction of PDS^(r)^ suture for the gastroenteric anastomosis. The reduction of stenosis was also verified along the development of this study from 2013, when routine usage of PDS 3-0^(r)^ suture for the gastrojejunal anastomosis performed over a Fouchet 32F^(r)^ probe, was implanted which ensured a minimal opening of 12 mm. The sample for the present study precedes this technical change and the suture used was Vicryl 3-0^(r)^.

Yang et al. presented marginal ulcer as the main finding in 31 of their patients (63.3%), and stenosis was the second finding (n=3)^18^. Similar data were mentioned by Wilson et al., with marginal ulcer affecting 81 patients and stenosis 29 (13%)^19^. Rocha obtained food impaction as the main alteration (n=18/8.3%), erosive esophagitis in second place (n=10/4.6%), followed by non-erosive gastritis (n=9/4.1%) and in fourth place, stenosis(n=5/2.3%)^21^. 

The stenosis of the anastomosis after bariatric surgery was also discussed by Obstein and Thompson^26 ^. The most common are the stenosis of the gastrojejunal anastomosis, as found in this study, with incidence in literature between 4-19%^27^^,^ 28 ^,^ 29 ^,^ 30. Espinel and Pinedo, state that the diagnosis occurs when there is resistance or impossibility to go through the gastrojejunal anastomosis with the endoscope, indicating a lumen smaller than 10 mm^31^. Schwartz et al. performed a prospective study with 1,000 patients focused only at the stenosis of the gastroenteric anastomosis after bypass, and found a prevalence of 3.2%^32^. Ramos et al. with 12,000 patients found a prevalence of 2.5% after bypass and suggest that the bigger the sample is, the smaller the rate of stenosis, with a result, in theory, closer to the general population who underwent this technique^25^.

To improve the understanding of the physiopathology of the mechanism of formation of the stenosis of gastrojejunal anastomosis, was analyzed if any preoperative variable would statistic significantly increase the chance of developing stenosis in the postoperative period. Was checked the variables gender, BMI, diabetes mellitus type 2 or reduced tolerance to glucose, dyslipidemia, preoperative endoscopic presence of hiatal hernia, erosive esophagitis, non-erosive esophagitis, erosive gastritis, non-erosive gastritis and if there would be a greater chance in case the preoperative upper endoscopy presented any alteration. But, the statistical analysis concluded that none of the preoperative factors that were studied had statistic significance to the increase of stenosis in the postoperative period of Roux-en-Y gastrojejunal bypass. This result corroborates with the consensual opinion of the surgeons, that stenosis is a complication which depends much more on the surgical technique than the particular variables of each patient^33^.

## CONCLUSIONS

The correlation between pre and postoperative findings of the upper digestive endoscopies indicated a reduction of hiatal hernia, erosive esophagitis, non-erosive esophagitis, erosive gastritis and non-erosive gastritis in the postoperative period. The stenosis of the gastrojejunal anastomosis was the most prevailing complication during the postoperative period with no correlation to the studied preoperative variables.
